# Influence of Impaired Upper Extremity Motor Function on Static Balance in People with Chronic Stroke

**DOI:** 10.3390/s24134311

**Published:** 2024-07-02

**Authors:** Ana Mallo-López, Alicia Cuesta-Gómez, Teresa E. Fernández-Pardo, Ángela Aguilera-Rubio, Francisco Molina-Rueda

**Affiliations:** 1 International Doctorate School, Rey Juan Carlos University, 28933 Madrid, Spain; 2 Department of Physiotherapy, Faculty of Sport Sciences, Universidad Europea de Madrid, Villaviciosa de Odón, 28670 Madrid, Spain; teresa.fernandez@universidadeuropea.es; 3 Department of Physical Therapy, Occupational Therapy, Rehabilitation and Physical Medicine, Faculty of Health Sciences, Rey Juan Carlos University, 28922 Madrid, Spain; 4 Motion Analysis, Ergonomics, Biomechanics and Motor Control Laboratory (LAMBECOM), Department of Physical Therapy, Occupational Therapy, Rehabilitation and Physical Medicine, Faculty of Health Sciences, Rey Juan Carlos University, 28922 Madrid, Spain; 5 Physiotherapy Department, Ramón y Cajal University Hospital, 28034 Madrid, Spain

**Keywords:** arm, balance, posturography, stance, stroke, upper extremity, Wii Balance Board

## Abstract

Background: Stroke is a leading cause of disability, especially due to an increased fall risk and postural instability. The objective of this study was to analyze the impact of motor impairment in the hemiparetic UE on static balance in standing, in subject with chronic stroke. Methods: Seventy adults with chronic stroke, capable of independent standing and walking, participated in this cross-sectional study. The exclusion criteria included vestibular, cerebellar, or posterior cord lesions. The participants were classified based on their UE impairment using the Fugl-Meyer Assessment of Motor Recovery after Stroke (FMA-UE). A posturographic evaluation (mCTSIB) was performed in the standing position to analyze the center of pressure (COP) displacement in the mediolateral (ML) and anteroposterior (AP) axes and its mean speed with eyes open (OE) and closed (EC) on stable and unstable surfaces. Results: A strong and significant correlation (*r* = −0.53; *p* < 0.001) was observed between the mediolateral (ML) center of pressure (COP) oscillation and the FMA-UE, which was particularly strong with eyes closed [*r*(EO) = 0.5; *r*(EC) = 0.54]. The results of the multiple linear regression analysis indicated that the ML oscillation is influenced significantly by the FMA-Motor, and specifically by the sections on UE, wrist, coordination/speed, and sensation. Conclusions: The hemiparetic UE motor capacity is strongly related to the ML COP oscillation during standing in individuals with chronic stroke, with a lower motor capacity associated with a greater instability. Understanding these relationships underpins the interventions to improve balance and reduce falls in people who have had a stroke.

## 1. Introduction

Stroke is one of the leading causes of disability today, with over 80% of patients requiring care and/or rehabilitation. Of relevance is the impact on balance, as a high percentage of people who have had a stroke fall, leading to impaired independence in activities of daily living due to postural instability. Therefore, understanding balance in people with chronic stroke is a major goal of neurorehabilitation [1].

Maintaining an upright posture requires multiple sensorimotor processes and sensory integration [2]. Somatosensory, vestibular, and visual information are required for postural orientation and are integrated by different neural networks [3]. In addition, the central nervous system (CNS) elaborates neuromuscular responses adapted to the context influenced by the musculoskeletal system [4]. Consequently, postural instability in people with chronic stroke may have multiple causes due to the involvement of multiple systems [5].

Several methods are currently available to assess balance, including validated clinical scales that provide performance information and instrumental methods that provide insight into the quality and neurophysiological strategies underlying postural control [6]. Posturography, which analyzes parameters such as the position of the center of pressure (COP), its oscillations in different axes, mean velocity, and the ability of the central nervous system to compensate for the suppression of sensory information, is one of the most widely used tools [7].

In posturography, the differences between healthy subjects and people who have had a stroke are significant. Individuals with hemiparesis employ adaptive strategies to enhance their sensorimotor abilities for effective execution; consequently, mimicking the patterns of healthy individuals without considering these adaptations is inadequate for the recovery of patients with stroke [8]. Therefore, studying the factors that influence postural stability in people who have had a stroke is highly relevant.

The LEs play a crucial role in locomotion and maintaining postural balance. The muscles and joints of the legs are essential for supporting the body weight, maintaining the center of gravity, and making the postural adjustments necessary for stability in both static and dynamic positions. For these reasons, there has traditionally been a focus on studying the effects of the LEs on balance.

Numerous studies have investigated the role of the lower extremities (LEs) in maintaining an upright posture in people with stroke using posturography. Motor impairment of the hemiparetic leg has been shown to affect the static balance, resulting in an asymmetry in the LE load [9], often compensated for by the increased use of the less affected leg and visual cues [10]. Particularly relevant is the contribution of the ankle strategy, which is often altered in patients with stroke, disrupting the shift of the center of pressure (COP) along the sagittal plane [11]. However, posturographic studies have shown that the main difference compared to healthy subjects is the increased displacement along the ML axis, although the contribution of the LEs in this direction is much smaller, involving the hip strategy [10]. Thanks to these studies and the integration of the pendulum model of balance [12], consisting of the lower limbs, trunk, and head, numerous treatments have been developed to improve the balance and gait of patients with stroke, focusing their intervention on the lower limbs and trunk [13,14].

The role of the UEs was studied later and mainly in situations in which they are in motion [15], such as reaching movements [16], or when their mobility is modified using slings [17]. In these cases, it has been shown how the paretic movement of the UEs produces greater changes in the movements of the trunk, resulting in greater postural instability, which people with stroke counteract with different strategies for shifting the COP in the AP and ML axes, depending on the task. Indeed, Rafsten et al. identified the predictive nature of arm motor deficiency in balance deficits in patients with chronic stroke [18]. Thanks to this knowledge, training reaching movements and repeating motor tasks of the affected upper limb are used to improve the dynamic balance of patients [19]. However, we do not yet fully understand the influence of the hemiparetic arm on performances in which the arm is not directly involved, such as static balance.

Given the important role of the arm and hand as sources of sensory information in the construction of the body schema and peripersonal space [20] and in the postural adjustments [21], we believe it is relevant to investigate the potential effects of sensorimotor impairment of the affected UE in a bipedal position. We believe that further investigation into the role of the upper limb in maintaining a bipedal posture may lead to new therapeutic approaches to improve balance in patients with stroke. This would not only help to maintain stance but could also improve performance in many basic daily activities that require postural orientation and adaptability.

Therefore, the aim of the present study was to analyze the effect of motor impairment of the hemiparetic UE on static standing balance in subjects with chronic stroke.

## 2. Materials and Methods

### 2.1. Study Design

This is a multicenter descriptive cross-sectional study involving subjects with chronic stroke in the Madrid region. The following centers participated in this study: Hospital Ramón y Cajal, “Centro de fisioterapia neurológica NeuroAvanza”, “Asociación de afectados por el ictus Rehabictus”, “Fundación Polibea”, “Hospital San Vicente”, “Centro “Téxum Fisioterapia”, and “Centro de Terapia Especializada en Neurología TEN”.

This study was approved by the local Research Ethics Committee (12/01/2020, registration number 0112202022320) and the reference hospital (28/09/21 ACTA 419) in accordance with the ethical principles of the Declaration of Helsinki. The authors report there are no competing interests to declare.

Subjects were recruited if they were over 18 years of age, had a non-recurrent stroke of more than 6 months’ duration, were able to stand upright for 60 s, walk without technical aids or orthoses in a controlled environment (functional ambulatory categories, FAC ≥ 3) [22], had no vestibular, cerebellar or spinal cord injury (Romberg negative) [23], and were able to understand and follow simple commands (Mini-Mental Test ≥ 23) [24]. Subjects had no previous functional impairment or neurological changes. Subjects were excluded if they had visual and/or hearing impairments that could not be corrected with ocular and/or hearing aids, osteoarticular pathology, or recent surgery. Subjects who had received botulinum toxin treatment one month before the assessment were also excluded. All participants signed the informed consent form after being informed verbally and in writing about the characteristics of this study.

### 2.2. Sample Size Estimation

The sample size was calculated using G*Power software (G*Power version 3.1.9.2). The following parameters were set retrospectively to obtain the sample size using a correlation model: two-tailed, expected correlation ρ H1 of 0.51, alpha error of 0.05, power of 0.80, and ρ H0 of 0.2, resulting in a required sample size of 63 participants.

### 2.3. Procedure

For recruitment, the participating centers provided access to their databases to contact subjects with chronic stroke who met the previously mentioned medical requirements. In an initial phone interview, the subjects were asked whether they could stand and walk without technical assistance in controlled environments. Those who answered affirmatively were scheduled for a single in-person evaluation by an expert physiotherapist in adults with neurological pathology. Only the subjects meeting all the inclusion criteria and no exclusion criteria during the in-person evaluation were included.

Initially, data were collected on the patient’s rehabilitation treatment, and various clinical tests of balance, motor function, and spasticity of the UE were performed to define the sample.

**Figure 1:**
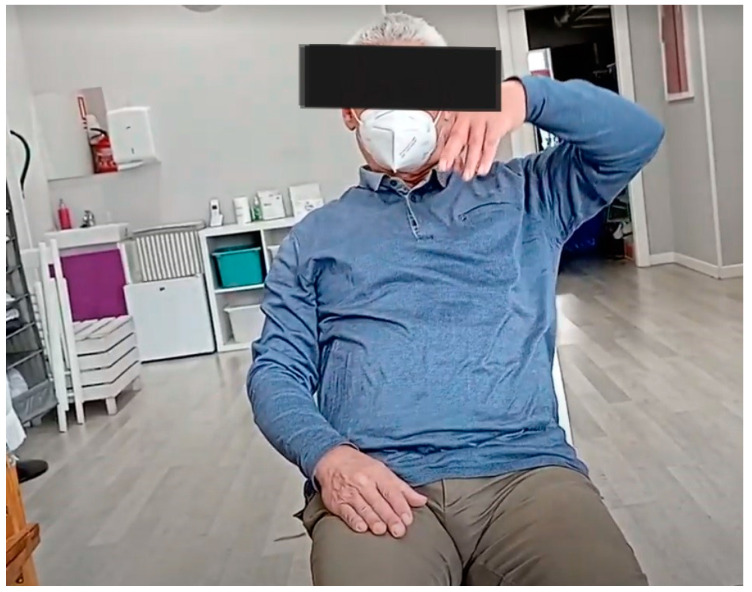
Participant performing the FMA-UE assessment.

The Berg Balance Scale (BBS) [25] and the Timed Up and Go test [26] were used to assess balance. The motor function of the UE was assessed using the Fugl-Meyer Assessment of Motor Recovery after Stroke (FMA-UE) [27]. This test assesses different components involved in the functionality of the UE in subsections, with a score for each: upper extremity (FMA-A), wrist (FMA-B), hand (FMA-C), coordination and speed (FMA-D), sensation (FMA-H), passive joint movement (FMA-I), and joint pain (FMA-J). The spasticity of the shoulder, elbow, and wrist flexors muscles was also assessed using the Modified Ashworth Scale, and data were recorded on the subject’s rehabilitation treatment: weekly frequency, intensity (duration of each session), and modality (group or individual) [28]. See Figure 1.

Second, the posturographic test was performed. The posturographic analysis of static balance was performed using the Modified Clinical Test of Sensory Interaction on Balance (mCTSIB) of the posturography program, which has been validated for people who have had a stroke using the Wii Balance Board^®^ (WBB) as a force platform [29]. The Posturography software is accessible and free of charge and can be used on any computer. Bluetooth is required to connect the computer with the WBB. Its features make it easy to transport and facilitate the participation of different centers.

The mCTSIB is a simplified version of the Sensory Organization Test that provides a quantitative assessment of an individual’s ability to maintain postural stability while standing in four conditions: open eyes (OEs) and closed eyes (CEs) on a stable surface and on an unstable surface (foam). The outcome measures of the test are the mean COP velocity and its oscillation in the anteroposterior (AP) and mediolateral (ML) axes for each condition [29].

During the posturography analysis, the participant must stand on the platform without any external aids in front of the computer screen, which is placed at eye level, and follow the instructions on the screen, which are read aloud by the physiotherapist. The physiotherapist helps the participant, if necessary, to step onto the platform and place each foot 10 cm from the center line of the platform, which is marked with colored lines. It is also explained to the participant that it is not allowed to move or separate the feet from the surface. The subject is first asked to stand on the platform for 30 s, as still as possible, with eyes open, staring at a point in the center of the screen. The same procedure is then repeated, but with eyes closed, and the beginning and end of the 30 s are indicated by an acoustic signal. The participant then steps off the platform and a foam rubber rectangle of size 46 × 29 × 8 cm and density 25 kg/m^3^ is placed on it, the participant steps onto the foam and repeats the previous tests. Each test was repeated three times [29].

**Figure 2:**
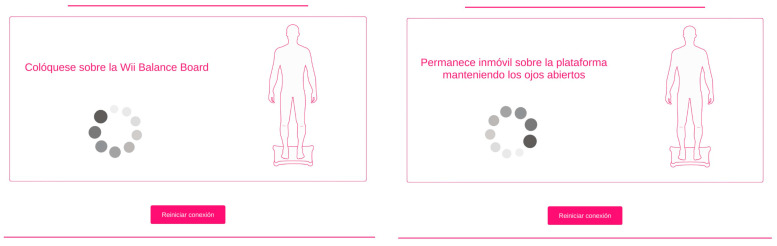
mCTSIB test instructions.

**Figure 3:**
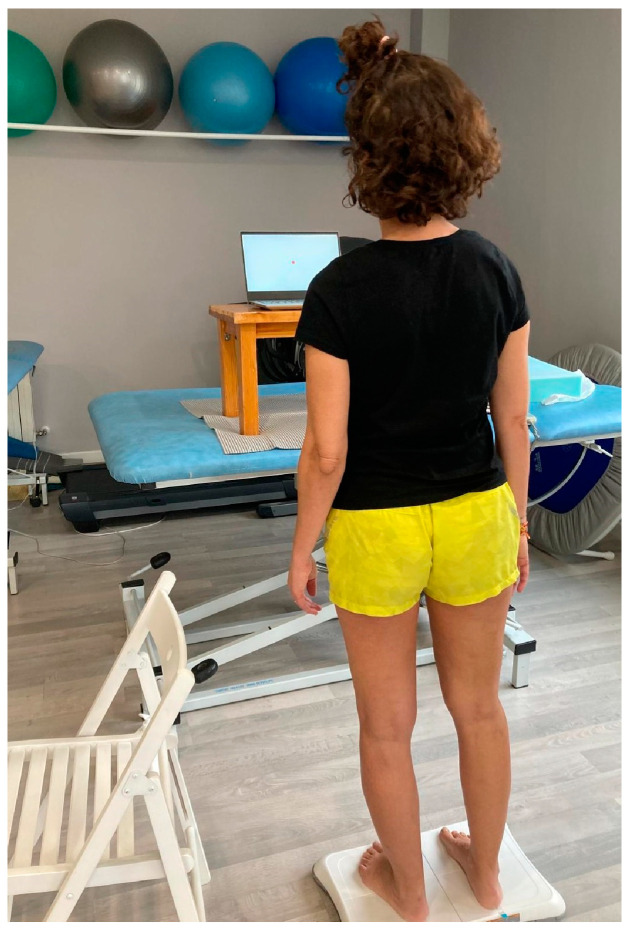
Participant performing mCTSIB test.

The participant was always accompanied by the physiotherapist, and the room was adapted beforehand to ensure patient safety and to prevent falls. See Figure 2 and Figure 3.

### 2.4. Statistical Analysis

Statistical analysis was performed using SPSS software for Windows, version 22.0 (IBM^®^ SPSS^®^ Statistics 22).

Descriptive analysis was used for all the variables. The quantitative variables were expressed as mean and standard deviation (SD). The qualitative variables were expressed as frequencies and percentages. The data were checked by histograms and quantile–quantile plots to see whether they were normally distributed.

A correlation analysis was carried out between the posturography variables and the UE motor function. For this purpose, Pearson’s correlation coefficient (*r*) was used. The correlation values were interpreted as small (*r* < ±0.29), medium (*r* = ±0.30–0.49), or strong (*r* = ±0.50–1.0) [30]. The Bonferroni correction was used to reduce the chance of coincidentally finding statistically significant results [31]. Therefore, the results were perceived as statistically significant when the *p* value was below 0.0041.

Based on the results of the correlations, to predict the influence of the UE motor function on the ML stability, multiple linear regressions using the Enter method were performed. For each condition of ML oscillation, six regression models were performed because of the six correlated categories of the FMA-UE. Age and injury time were handled as possible confounders and were adjusted for in the regression models. The Bonferroni correction was used; therefore, the outcomes were perceived as statistically significant when the *p* value was below 0.0083.

## 3. Results

The initial sample comprised 70 subjects, but 5 were excluded; 3 due to botulinum toxin treatment and 2 because of recent surgery. The final sample consisted of 65 participants, whose characteristics are shown in Table 1. The scoring of the different tests is available in the Supplementary File (Table S1) The results of correlations between the posturographic values of static balance and the UE motor function are presented in Table 2, Table 3 and Table 4. Table 5 shows the significant data obtained in the regression models.

### 3.1. Correlation between mCTSIB and FMA-UE

As shown in Table 2, a strong correlation (*r* = −0.53; *p* < 0.001) was observed between the ML COP oscillation with CEs and the FMA-Motor, with moderate significant correlation for the other conditions. Additionally, a significant and strong correlation was found between the FMA-A and the ML COP oscillation across all conditions except for OEs with foam (*r* = −0.43), with stronger correlations noted for eyes-closed conditions [*r*(OEs) = −0.5; *r*(CEs) = −0.54; *r*(CEs foam) = −0.53]. Similarly significant but moderate correlations were observed for FMA-D and FMA-H, with correlation coefficients (*r*) ranging from −0.35 to −0.49. For FMA sections B–C, there were significant moderate correlations only for the eyes-closed conditions, whereas in sections J and I, no correlations were observed.

These results suggest that UE motor impairment is associated with COP instability in the frontal plane, particularly under conditions with CEs. Specifically, a stronger correlation was observed between the impairment of arm motor function, UE coordination and sensation. Hand motor capacity (except for eyes closed on a stable surface), pain, and passive joint ranges were not associated with increased frontal instability. No correlations were found between UE motor capacity and COP oscillation in the AP axis.

### 3.2. Multiple Linear Regression to Assess the Influence of FMA-UE on Balance Parameters

Table 3 presents the outcomes derived from multiple linear regression models assessing the impact of arm function capacity on ML COP oscillation. All the balance metrics under investigation were significantly influenced by five out of the six FMA-UE categories, after adjusting for age and time since lesion to account for potential confounding factors. The results indicated that the ML oscillation was influenced significantly by the FMA-Motor, and all its subsections except the one studying hand function. UE sensation also influenced the COP oscillation. The standardized β coefficient was utilized to quantify the magnitude of influence on the balance parameters. No multicollinearity issues were detected based on variance inflation factor criteria (1 < VIF < 2). The first model, studying UE global motor function (FMA-Motor), age, and injury time, explained 31.2% of the COP oscillations variability along the ML axis under CEs conditions [β(FMA-Motor) = −0.560, *p* ≤ 0.001 *; β(age) = −0.089, *p* = 0.412; β(injury time) = −0.135, *p* = 0.227)] and 20.3% under OE conditions [β(FMA-Motor) = −0.441, *p* ≤ 0.001 *; β(age) = −0.030, *p* = 0.798; β(injury time) = 0.027, *p* = 0.819)]. For the rest of the models, the age and years of injury followed the same pattern, not significantly influencing the balance variables.

These results demonstrate the impact of the UE motor function on the frontal plane COP movement: the greater the UE impairment, the greater the postural instability. Specifically, the models that showed the greatest influence on frontal instability were those that looked at arm dysfunction (FMA-A), impaired coordination (FMA-D), and sensation (FMA-H), in that order of importance.

## 4. Discussion

To the best of our knowledge, no previous studies have analyzed the relationship between the motor deficit of the hemiparetic UE and the static balance assessed by posturography. Hemiplegia is the most common sensorimotor sequela after stroke, resulting in frontal plane asymmetry. As a result, the CNS prioritizes stability control along the ML axis, using different strategies depending on the areas affected [32]. Interventions targeting components that influence frontal stability in patients with stroke could significantly improve their balance and functionality. The results of our study appear to be the first to show a significant strong negative correlation between the FMA-UE and COP oscillation control in the ML axis during standing in participants with chronic stroke: the more severe the motor impairment of the UE, the greater the instability in the frontal plane. These findings highlight the importance of investigating the role of the hemiparetic UE in static balance.

Qin et al. demonstrated that the arm plays a role in maintaining upright posture in people who have had a stroke, as standing increased spasticity in the UE flexors [33]. The authors suggested that increased UE spasticity may be a compensatory strategy of the CNS to increase postural stability, highlighting the involvement of the UE in postural control. In addition, a light touch of the UE is known to reduce postural sway in the ML axis, ref. [34], highlighting the importance of tactile function in modulating cortical representation and promoting balance by activating the prefrontal cortex [35].

Frontal plane stability is a significant challenge for people who have had a stroke [10], and further investigation into mechanisms to improve it is warranted. Numerous studies have investigated LE strategies, highlighting the role of the ankle strategy for swing control in the AP axis and the hip strategy for the ML axis [36]. However, some research suggests that the contribution of the LE to frontal plane motor function in maintaining a standing posture in people who have had a stroke is minimal [37]. Conversely, studies have highlighted the importance of the upper limbs for frontal plane stability during postural stability tasks [38]. Our findings are consistent with these studies, as no significant relationships were observed between FMA-UE and COP oscillation in the AP axis, consistent with the greater influence of the LE. Furthermore, participants in our study had a low risk of falling (mean Berg Balance Scale score of 50.34, SD ± 4.819) and demonstrated the ability to stand and walk without assistive devices, indicating proficiency in LE and trunk control. Therefore, the degree of UE involvement may serve as a differentiating factor contributing to different degrees of frontal plane stability.

Johnson et al. found that older adults rely more on upper body movements to maintain postural control, especially when sensory feedback is compromised. They showed a greater center of pressure (COP) range with restricted arm movements, particularly in the frontal plane. In contrast, young adults showed an increased ML displacement under restricted arm movements only in the most difficult sensory conditions [39]. These results are consistent with our findings. In patients with stroke with altered or impaired sensory information, the reduced motor capacity of the arm may hinder the development of efficient compensatory strategies. This confirms that greater UE impairment is associated with increased frontal instability.

However, other studies in stroke populations have shown that training in arm reaching movements reduces center of pressure (COP) shifts not only in the mediolateral (ML) axis but also in the anteroposterior (AP) axis [40]. Purposeful arm movements induce shifts in the body’s centre of mass, resulting in greater COP displacement in both axis when using the affected UE, employing diverse patterns and strategies depending on the impairment [41]. Based on the knowledge of motor learning, involving the upper limb in certain tasks could enhance the CNS’s ability to calibrate, for example by producing postural adjustments that improve balance [42]. Investigating this novel approach and incorporating it into rehabilitation treatment may be of considerable interest. UE therapeutic approaches based on reaching exercises in patients with stroke to improve postural control are consistent with our results, as the correlation between COP oscillation was stronger for FMA-Motor and FMA-A. Several studies have highlighted the importance of scapular stability and reaching patterns in improving balance and postural stability [19,43]. Nevertheless, in our knowledge there is no evidence supporting the significance of the upper limb in the sagittal plane during a static stance.

In addition, the implications of balance in individuals with UE amputation have been studied, indicating how the absence of the limb or even the use of a prosthesis increases instability, highlighting the importance of internal models of the limb in balance [44]. People who have had a stroke also exhibit alterations in these internal models of the UE, albeit due to entirely different causes. Moreover, stability control in the ML plane is more severely affected in subjects with stroke due to alterations in postural and visual vertical perception [45]. The somatosensory system plays a crucial role in the construction of vertical perception, and people who have had a stroke often have difficulties in accurately receiving and integrating somatosensory information due to cortical lesions [45]. The UE is extensively represented in the parietal cortex due to its high density of receptors and capacity for selective movement [46,47]. Previous research has shown that greater cortical representation of the hand after stroke correlates with improved functional recovery [48]. Recent studies have highlighted systematic biases in metric body representation and peripersonal space in patients with chronic stroke who have persistent sensorimotor deficits. In addition, authors have suggested that body segments, particularly the hand, disrupt the dynamic spatial mapping for perception and action while providing intrinsic spatial cues critical for somatosensory processing [49,50]. These findings may be relevant to our study, which suggests a greater relevance of the sensory aspect of the hand in standing balance compared to the motor aspect. Specifically, we found that FMA-H (sensation) significantly impacted the postural instability, whereas FMA-C (hand motor function) did not. In addition, participants in our study did not have visual, vestibular, cerebellar, or cognitive impairments, with only the somatosensory system being affected by the aftereffects of stroke, either directly through primary lesions in somatosensory cortical areas or indirectly through neural reorganization strategies [51]. Our findings support further research into the somatosensory performance of the UE, its role in the body schema and its influence on bipedal posture.

The relationship between COP oscillation in the ML axis and UE motor impairment is particularly pronounced with the eyes closed. In general, people who have had a stroke show greater COP oscillations with their eyes closed, especially in the ML plane, compared to healthy subjects [10]. Therefore, we hypothesize that a greater motor capacity of the affected UE contributes to better COP stability in the frontal plane under closed-eye conditions as indicated by the regression models analyzed in this study. In this scenario, in which visual dominance in postural control is not feasible, the relevance of vestibular (preserved in our sample) and somatosensory information increases. When somatosensory afferents from the LE are reduced (as in the foam condition of the test), the negative relationship between COP displacement in the frontal plane and sensory perception (section FMA-H) is strengthened. Thus, it is conceivable that sensory and alignment information received from the arm may help to improve stability in the ML axis when LE information is impaired [52,53]. Perhaps, protocols could be developed to improve postural orientation, which, therefore, would improve balance by addressing the UE sensory components, including its conscious and unconscious perception. Further research is needed.

Finally, interventions targeting components that influence frontal stability in patients with stroke could significantly improve their balance and functionality. This study highlights a robust correlation and influence between UE impairment and frontal stability. Functional MRI research identifies different recovery patterns between the UE and the LE after a stroke, necessitating the tailoring of neurorehabilitative protocols [54]. Our study highlights the need for further research into the cortical reorganization of the UE, not only in manual or grasping tasks but also in its role in postural maintenance. This research is crucial for the advancement and development of novel therapeutic strategies to improve postural control in patients with chronic stroke, thereby enhancing their quality of life.

This study has limitations that should be noted. Firstly, the sample characteristics are not homogeneous in terms of age and injury time. However, previous research has shown that age does not significantly affect the COP sway, suggesting that this heterogeneity is unlikely to bias the results [55]. In addition, both variables were included in the regression models without any change in the influence of the UE motor ability on the balance parameters. Secondly, it would have been interesting to include outcome measures of the UE activity, such as surface electromyography or functional magnetic resonance imaging of brain activity, but the costs associated with these instrumental systems led us to opt for a validated posturography test for chronic stroke, which is freely available and inexpensive. This decision was made to ensure ethical considerations and to avoid large budgetary investments without a prior understanding of the potential utility of the results.

## 5. Conclusions

Our study shows a strong correlation between the motor function of the hemiparetic UE and the COP oscillation in the ML axis during standing in subjects with chronic stroke. Greater UE impairment influences the COP frontal plane oscillation, increasing postural instability.

These findings highlight the importance of considering the role of the UE in frontal plane stability in people who have had a stroke and suggest potential therapeutic avenues to improve static balance in this population. Further research in this area may lead to the development of targeted interventions aimed at improving UE function to optimize postural control and reduce the risk of falls in people with chronic stroke.

## Figures and Tables

**Table 1:** Sample characteristics.

Parameters		
* Age (years)		56.77 (±12.860)
^†^ Sex (frequencies and percentages)		M: 37 (56.92%); F: 28 (43.08%)
* Injury time (years)		4.469 (±4.8169)
^†^ Affected side		R: 27 (41.54%); L: 38 (58.46%)
^†^ Stroke type		Ischemic: 45 (69.23%); Haemorrhagic: 20 (30.77%)
* TUG		12.4035 (±7.23)
* BBS		50.34 (±4.819)
^†^ FAC		5: 36 (55.38%); 4: 23 (35.39%); 3: 6 (9.23%)
^†^ Individual treatment		32 (49.23%)
^†^ Groupal treatement		11 (16.92%)
^†^ Absence of treatment		22 (33.85%)
* Teatment intensity (hours/week)		0.6769 (±0.57405)
^†^ MAS	Shoulder flexors	1+: 3 (4.62%); 1: 19 (29.23%); 0: 43 (66.15%)
	Elbow flexors	4: 2 (3.08%); 3: 6 (9.23%); 2: 10 (15.38%); 1+: 11 (16.92%); 1: 9 (13.85%); 0: 27 (41.54%)
	Wrist flexors	4: 1 (1.54%); 3: 10 (15.38%); 2: 27 (10.78%); 1+: 2 (3.08%); 1: 11 (16.92%); 0: 34 (52.30%)

* Data expressed as mean standard deviation (SD). ^†^ Data expressed as frequencies and percentages. MAS: Modified Ashworth Scale, FAC: functional ambulation category, TUG: Timed Up and Go test, BBS: Berg Balance Scale.

**Table 2:** Correlation between mCTSIB and FMA-Motor total (A–D).

Balance Parameters	FMA-Motor (A–D)			
	*r*	*CI* 95%		*p* Value
		*UL*	*LL*	
mCTSIB oscillation AP OEs (cm)	0.073	0.82	0.59	0.566
mCTSIB oscillation AP CEs (cm)	0.021	0.43	−0.03	0.867
mCTSIB oscillation AP OEs foam (cm)	−0.070	−0.55	−0.80	0.582
mCTSIB oscillation AP CEs foam (cm)	−0.24	0.00	−0.46	0.058
mCTSIB oscillation ML OEs (cm)	−0.44	−0.23	−0.62	<0.001 *
mCTSIB oscillation ML CEs (cm)	−0.53	−0.33	−0.68	<0.001 *
mCTSIB oscillation ML OEs foam (cm)	−0.39	−0.16	−0.58	0.001 *
mCTSIB oscillation ML CEs foam (cm)	−0.49	−0.28	−0.66	<0.001 *
mCTSIB mean speed OEs (cm/s)	−0.22	0.02	−0.44	0.076
mCTSIB mean speed CEs (cm/s)	−0.28	−0.03	−0.49	0.023
mCTSIB mean speed OEs foam (cm/s)	−0.07	0.17	−0.30	0.582
mCTSIB mean speed CEs foam (cm/s)	−0.39	−0.16	−0.58	0.001 *

*r*: Pearson’s correlation coefficient, *CI*: confidence interval, *UL*: upper limit, *LL*: lower limit, mCTSIB: Modified Clinical Test of Sensory Interaction on Balance, FMA: Fugl-Meyer Assessment, AP: anteroposterior, ML: mediolateral, OEs: open eyes, CEs: closed eyes, * *p* value ≤ 0.0041.

**Table 3:** Correlation between mCTSIB and FMA-A, FMA-B, FMA-C, and FMA-D.

Balance Parameters	FMA-A				FMA-B				FMA-C				FMA-D			
	*r*	*CI* 95%		*p* Value	*r*	*CI* 95%		*p* Value	*r*	*CI* 95%		*p* Value	*r*	*CI* 95%		*p* Value
		*UL*	*LL*			*UL*	*LL*			*UL*	*LL*			*UL*	*LL*	
mCTSIB oscillation AP OEs (cm)	0.05	0.29	−0.19	0.647	0.049	0.65	0.27	0.697	0.10	0.33	−0.14	0.430	0.02	0.26	−0.22	0.849
mCTSIB oscillation AP CEs (cm)	0.01	0.25	−0.23	0.923	0.00	0.24	−0.24	0.986	0.06	0.29	−0.18	0.602	−0.02	0.22	−0.26	0.827
mCTSIB oscillation AP OEs, foam (cm)	−0.13	0.11	−0.36	0.288	0.00	0.24	−0.24	0.997	0.04	0.28	−0.20	0.751	−0.15	0.09	−0.37	0.208
mCTSIB oscillation AP CEs foam (cm)	−0.28	−0.03	−0.49	0.022	−0.21	0.03	−0.43	0.085	−0.08	0.16	−0.31	0.491	−0.23	0.01	−0.44	0.070
mCTSIB oscillation ML OEs (cm)	−0.50	−0.29	−0.66	<0.001 *	−0.36	−0.12	−0.55	0.003 *	−0.29	−0.04	−0.49	0.016	−0.38	−0.15	−0.57	0.002 *
mCTSIB oscillation ML CEs (cm)	−0.54	−0.34	−0.69	<0.001 *	−0.43	−0.20	−0.60	<0.001 *	−0.44	−0.21	−0.61	<0.001 *	−0.48	−0.26	−0.64	<0.001 *
mCTSIB oscillation ML OEs, foam (cm)	−0.43	−0.20	−0.60	<0.001 *	−0.29	−0.04	0.49	0.018	−0.25	0.00	−0.46	0.041	−0.40	−0.17	−0.58	0.001 *
mCTSIB oscillation ML CEs, foam (cm)	−0.53	−0.32	−0.68	<0.001 *	−0.42	−0.19	−0.60	0.001 *	−0.32	−0.08	−0.52	0.008	−0.49	−0.27	−0.65	<0.001 *
mCTSIB mean speed OEs (cm/s)	−0.26	−0.01	−0.47	0.035	−0.15	0.09	−0.37	0.210	−0.12	0.12	−0.35	0.33	−0.25	0.00	−0.46	0.041
mCTSIB mean speed CEs (cm/s)	−0.32	−0.08	−0.52	0.007	−0.21	0.03	−0.43	0.091	−0.15	0.09	−0.37	0.211	−0.32	−0.08	−0.52	0.008
mCTSIB mean speed OEs, foam (cm/s)	−0.38	−0.15	−0.57	0.002 *	−0.25	0.00	−0.46	0.044	−0.22	0.02	−0.44	0.078	−0.35	−0.11	−0.54	0.004 *
mCTSIB mean speed CEs, foam (cm/s)	−0.32	−0.08	−0.52	0.009	−0.27	−0.02	−0.48	0.032	−0.18	0.06	−0.40	0.146	−0.31	−0.07	−0.51	0.013

*r*: Pearson’s correlation coefficient, *CI*: confidence interval, *UL*: upper limit, *LL*: lower limit, mCTSIB: Modified Clinical Test of Sensory Interaction on Balance, FMA: Fugl-Meyer Assessment, FMA-A: upper extremity, FMA-B: wrist, FMA-C: hand, FMA-D: coordination and speed, AP: anteroposterior, ML: mediolateral, OEs: open eyes, CEs: closed eyes. * *p* value ≤ 0.0041.

**Table 4:** Correlation between mCTSIB and FMA-H, FMA-I, and FMA-J.

Balance Parameters	FMA-H				FMA-I				FMA-J			
	*r*	*CI* 95%		*p* Value	*r*	*CI* 95%		*p* Value	*r*	*CI* 95%		*p* Value
		*UL*	*LL*			*UL*	*LL*			*UL*	*LL*	
mCTSIB oscillation AP OEs (cm)	−0.11	0.13	−0.34	0.364	0.12	0.35	−0.12	0.320	−0.06	0.18	−0.29	0.635
mCTSIB oscillation AP CEs (cm)	−0.19	0.05	−0.41	0.127	0.05	0.29	−0.19	0.676	−0.10	0.14	−0.33	0.409
mCTSIB oscillation AP OEs foam (cm)	−0.17	0.07	−0.39	0.154	0.07	0.30	−0.17	0.558	−0.08	0.16	−0.31	0.498
mCTSIB oscillation AP CEs foam (cm)	−0.15	0.09	−0.37	0.226	−0.13	0.11	−0.36	0.308	−0.20	0.04	−0.42	0.101
mCTSIB oscillation ML OEs (cm)	−0.35	−0.11	−0.54	0.003 *	−0.24	0.00	−0.45	0.046	−0.06	0.18	−0.29	0.612
mCTSIB oscillation ML CEs (cm)	−0.41	−0.18	−0.59	0.001 *	−0.25	0.00	−0.46	0.040	0.10	0.33	−0.14	0.426
mCTSIB oscillation ML OEs foam (cm)	−0.40	−0.17	−0.58	0.001 *	−0.22	0.02	−0.44	0.066	−0.02	0.22	−0.26	0.842
mCTSIB oscillation ML CEs foam (cm)	−0.49	−0.27	−0.65	<0.001 *	−0.28	−0.03	−0.49	0.024	−0.14	0.10	−0.37	0.275
mCTSIB mean speed OEs (cm/s)	−0.18	0.06	−0.40	0.147	0.00	0.24	−0.24	0.950	0.00	0.24	−0.24	0.978
mCTSIB mean speed CEs (cm/s)	−0.20	0.04	−0.42	0.103	0.29	0.49	0.04	0.819	−0.07	0.17	−0.30	0.551
mCTSIB mean speed OEs, foam (cm/s)	−0.31	−0.07	−0.51	0.011	0.00	0.24	−0.24	0.958	0.01	0.25	−0.23	0.918
mCTSIB mean speed CEs, foam (cm/s)	−0.16	0.08	−0.38	0.209	−0.04	0.20	−0.28	0.746	0.00	0.24	−0.24	0.979

*r*: Pearson’s correlation coefficient, *CI*: confidence interval, *UL*: upper limit, *LL*: lower limit, mCTSIB: Modified Clinical Test of Sensory Interaction on Balance, FMA: Fugl-Meyer Assessment, FMA-H: sensation, FMA-I: passive joint movement, FMA-J: pain, AP: anteroposterior, ML: mediolateral, OEs: open eyes, CEs: closed eyes. * *p* value ≤ 0.0041.

**Table 5:** Multiple regression models analysis.

	mCTSIB Oscillation ML OEs (cm)				mCTSIB Oscillation ML CEs (cm)				mCTSIB Oscillation ML OEs, Foam (cm)				mCTSIB Oscillation ML CEs, Foam (cm)			
	*R* ^2^	*B*	*SE*	*p Value*	*R* ^2^	*B*	*SE*	*p Value*	*R* ^2^	*B*	*SE*	*p Value*	*R* ^2^	*B*	*SE*	*p Value*
FMA A-D	0.203	−0.441	0.009	<0.001 *	0.312	−0.560	0.01	<0.001 *	0.165	−0.415	0.011	0.001 *	0.267	−0.524	0.012	<0.001 *
FMA-A	0.258	−0.497	0.017	<0.001 *	0.325	−0.564	0.019	<0.001 *	0.197	−0.450	0.021	<0.001 *	0.302	−0.552	0.022	<0.001 *
FMA-B	0.135	−0.354	0.047	0.006 *	0.218	−0.468	0.052	<0.001 *	0.093	−0.314	0.059	0.017	0.197	−0.453	0.063	<0.001 *
FMA-C	0.096	−0.282	0.034	0.028	0.174	−0.453	0.037	<0.001 *	0.073	−0.271	0.042	0.037	0.128	−0.354	0.046	0.006 *
FMA-D	0.150	−0.371	0.097	0.004 *	0.258	−0506	0.105	<0.001 *	0.178	−0.433	0.116	0.001 *	0.275	−0.535	0.123	<0.001 *
FMA-H	0.135	−0.343	0.057	0.006 *	0.184	−0.412	0.064	0.001 *	0.173	−0.418	0.067	0.001 *	0.269	−0.518	0.071	<0.001 *

mCTSIB: Modified Clinical Test of Sensory Interaction on Balance, FMA: Fugl-Meyer Assessment, FMA-A: upper extremity, FMA-B: wrist, FMA-C: hand, FMA-D: coordination and speed, FMA-H: sensation, ML: mediolateral, OEs: open eyes, CEs: closed eyes. * *p* value ≤ 0.0083.

## Data Availability

Data are contained within the article.

## Supplementary Materials

The following supporting information can be downloaded at: https://www.mdpi.com/article/10.3390/s24134311/s1, Table S1: Scores on clinical scales and instrumental tests.
