# Supplementary material for: Influence of Impaired Upper Extremity Motor Function on Static Balance in People with Chronic Stroke
Source: Sensors (Basel). 2024 Jul 2;24(13):4311. doi: 10.3390/s24134311 (PMC11244378; doi:10.3390/s24134311)
Supplement: Supplementary file 1 [file sensors-24-04311-s001.zip › sensors-3067534-supplementary.pdf]

Table S1: Scores on clinical scales and instrumental tests.

**Parameters**	
FMA motor (A-D)	39.05 (±18.19)
FMA A	21.94 (±9.18)
FMA B	4.97 (±3.64)
FMA C	8.34 (±5.10)
FMA D	3.85 (±1.73)
FMA H	8.12 (±2.93)
FMA I	20.98 (±2.57)
FMA J	22.25 (±3.00)
mCTSIB oscillation AP open eyes (cm)	3.13 (±1.51)
mCTSIB oscillation AP closed eyes (cm)	3.48 (±1.41)
mCTSIB oscillation AP open eyes foam (cm)	3.04 (± 1.39)
mCTSIB oscillation AP closed eyes foam (cm)	4.24 (±1.61)
mCTSIB oscillation ML open eyes (cm)	2.28 (±1.38)
mCTSIB oscillation ML closed eyes (cm)	2.58 (±1.59)
mCTSIB oscillation ML open eyes foam (cm)	2.86 (±1.67)
mCTSIB oscillation ML closed eyes foam (cm)	3.43 (±1.87)
mCTSIB mean speed open eyes (cm/s)	1.10 (±0.48)
mCTSIB mean speed closed eyes (cm/s)	1.64 (±0.84)
mCTSIB mean speed open eyes foam (cm/s)	1.69 (±0.91)
mCTSIB mean speed closed eyes foam (cm/s)	2.83 (±1.58)

Data expressed as mean standard deviation (SD), FMA: Fugl Meyer Assessment, FMA A: upper extremity, FMA B: wrist, FMA C: hand, FMA D: coordination and speed, FMA H: sensation, FMA I: passive joint movement, FMA J: pain, mCTSIB: Modified Clinical Test of Sensory Interaction on Balance, AP: anteroposterior, ML: mediolateral.
